# A pilot randomized controlled trial to examine the impact of a therapy dog intervention on loneliness in adult patients hospitalized in a psychiatric unit

**DOI:** 10.3389/fpsyt.2025.1582767

**Published:** 2025-12-10

**Authors:** Nancy R. Gee, Lisa Townsend, Erika Friedmann, Sandra B. Barker, Tushar P. Thakre, Megan K. Mueller

**Affiliations:** 1 Center for Human-Animal Interaction, Department of Psychiatry, School of Medicine, Virginia Commonwealth University, Richmond, VA, United States; 2 Department of Organizational Systems and Adult Health, University of Maryland School of Nursing, Baltimore, MD, United States; 3 Department of Psychiatry, School of Medicine, Virginia Commonwealth University, Richmond, VA, United States; 4 Department of Comparative Pathobiology, Cummings School of Veterinary Medicine, Tufts University, North Grafton, MA, United States

**Keywords:** animal-assisted intervention, loneliness, well-being, therapy dog, psychiatric in-patients

## Abstract

**Background and objectives:**

Loneliness has been linked to a number of health threats and is associated with significant morbidity and mortality risks among people with mental illness. Loneliness fully mediates the relationship between societal stigma and depression as well as thoughts of self-injury and is a precursor to suicide attempts and relapse in people with substance misuse disorders.

**Research design and methods:**

Individuals hospitalized for the treatment of mental illness (N = 60) were randomly assigned to one of three conditions: dog + handler intervention (AAI), handler only conversational control (CC), or usual care (UC) for 20 minutes per day for three days. An Analog scale and two versions of the UCLA loneliness scale (UCLA-SF and UCLA-LS) were used to assess loneliness. Linear Mixed Models with random intercepts were applied to compare changes in loneliness between conditions pre/post daily intervention sessions, pre/post 3-day intervention phase (days 2–4), and baseline to post-intervention (days 1–5).

**Results:**

Loneliness decreased significantly more in the AAI group relative to the CC or UC groups in daily pre/post intervention sessions, and over the course of the 3-day intervention for all participants (*p <*.01). Within those results nuanced male/female sex differences and dog ownership status differences are reported. In stratified analysis, reduction in loneliness for the AAI group occurred from baseline to follow-up, but only among dog owners (*p <*.01).

**Discussion and implications:**

These results indicate that AAI was effective for reducing loneliness in people who are hospitalized for the treatment of mental illness. These results support and extend previous research and suggest that AAI has the potential to be effective as an adjunctive treatment for loneliness among people hospitalized for mental illness.

**Clinical Trial Registration:**

clinicaltrials.gov identifier, NCT05089201.

## 1 Introduction

Loneliness is linked to a number of health threats, is considered to be as harmful as smoking 15 cigarettes a day, and is potentially more dangerous than obesity (1). Loneliness was considered to be at epidemic proportions (2) prior to the COVID-19 pandemic. The resulting need for social distancing during the pandemic exacerbated loneliness and other mental health concerns that continue into the post-pandemic era.

Loneliness is associated with significant morbidity and mortality risks among people with mental illness. As many as 70% of community-dwelling adults with mental illness endorse experiencing loneliness (3), which increases exponentially with the number of co-morbid psychiatric diagnoses (4). In older adult psychiatric inpatients, loneliness has a strong relationship with symptoms of depression, mental illness severity, and poorer well-being (5). The subjective experience of loneliness fully mediates the relationship between societal stigma and depression (6) as well as thoughts of self-injury (7) and is a precursor to substance abuse relapse in adults with dual mental health and substance misuse disorders (8). Adults with mental illness who report loneliness face increased odds of psychiatric hospitalization (9, 10). Phenomenological studies identify loneliness and isolation as two of the highest-ranked unmet needs in this population (11) which represent key factors in the decision to attempt suicide (12). Prospective studies provide the gravest evidence regarding loneliness and mental illness: a meta-analysis of 17 longitudinal studies indicates that people who experience loneliness remain at heightened risk of suicide attempt up to five years after assessment (13).

Accumulated evidence supports that companion animals offer a number of health and wellbeing benefits (14, 15). Interacting with a trained therapy animal as part of an animal-assisted interaction (AAI) may provide social support for patients with mental illness, thus reducing the risk of loneliness. AAI has been leveraged as an intervention to improve social functioning in older psychiatric (16) and dementia patients (17, 18) and reported to reduce anxiety (19–21), depression (21), pain (21), and fear (22) in hospitalized patients with mental illness. Studies also report an association between AAI and increased attendance in group therapies (23, 24) and adherence to treatment (25) in hospitalized patients with serious mental illness Focusing specifically on AAI and patients with schizophrenia, studies report improved physiological stress levels, treatment adherence, symptomatology (26, 27), self-esteem, self-determination (27), and reduced positive and negative psychotic symptoms (28).

These indications that interacting with therapy animals can ameliorate loneliness and other psychiatric symptoms are encouraging, but there is little research into the qualities of the most effective interactions. In particular, the use of AAIs for hospitalized patients with mental illness as a strategy for reducing loneliness has been understudied. Scientists and practitioners need clear answers to some key questions before specific recommendations can be made. For example, there are relatively few studies of AAI that focus on AAI as an adjunctive approach for addressing loneliness and health-related outcomes in hospital settings. In fact, we are aware of only one such study. A recent study of hospitalized older adults (29) implemented a randomized control trial in which patients were randomly assigned to one of three conditions; 1) AAI – visits from a therapy dog and their handler, 2) Conversational Control (CC) – visits with the handler only, and 3) Usual Care (UC) in an inpatient hospital setting. The study took place over 5 days, in which day 1 implemented enrollment and baseline measures, days 2–4 were the intervention days, and day 5 included follow-up measures. The results showed that AAI was effective for reducing loneliness in hospitalized older adults when compared with UC, but the CC condition was not. That study showed that there is something unique about the presence of a dog, above the contribution of their handler, in reducing loneliness for hospitalized older adults. No such study has examined loneliness in hospitalized psychiatric patients. The purpose of the current study was to replicate and extend these findings to an inpatient psychiatry population using the same measures and methodology. Based on previous research (e.g., 29) we predict immediate reductions in loneliness for those patients in the AAI group relative to UC, but similar reductions are not likely to occur in the CC condition. We recognize that psychiatric inpatients may be differentially responsive to the presence of the dog and their handler, necessitating this study to address this issue.

## 2 Methods

### 2.1 Ethics approvals

This study was reviewed and approved by the Virginia Commonwealth University Institutional Review Board (HM20021567). The Virginia Commonwealth University Institutional Animal Care and Use Committee deemed the study exempt because the dogs involved in this study were privately housed and owned and were not subjects of the investigation. The study was also reviewed by the Local Human Rights Committee (LHRC) and was acknowledged to be implemented in accordance with the Human Rights Regulations.

### 2.2 Design

In this randomized controlled trial participants were randomly assigned to one of three treatment conditions: 1) AAI (Animal-Assisted Intervention) – visits from a therapy dog and their handler, 2) CC (Conversational Control) – visits from the handler without their dog, or 3) UC (Usual Care) – usual care in a hospital setting. Each participant received one condition, thus the independent variable was delivered between subjects, but participants were also assessed repeatedly over the course of the 3-day intervention and at 1- and 6-month follow-up, such that repeated measures were also captured. The primary outcome variable was loneliness.

The study was conducted in three phases: day 1 (screening/baseline) and days 2 to 4 (intervention delivery), and day 5 (post-intervention). There were also two follow-up time points (1 and 6-months).

The sample of 60, 20 participants in each group of the pilot study, was based on *a priori* power analysis with Cohen’s d=0.25, α=0.05, and β=0.80 with estimated unconditional interclass correlations of 0.7 based on our experience of very high intercorrelations among repeated measures of mood over several weeks. Since this is a pilot study, the ranges, variability, and intercorrelations of values for the outcomes in this population are among the aims of the study. This pilot study sample of 20 per group provides information that will allow better refinement of sample size calculations for a larger, outcome focused randomized clinical trial (30).

Random assignment to conditions was generated using randomizer.org. Participants were stratified based on dog ownership status (non-owners vs. owners) and were randomly assigned to one of the three conditions in blocks of six. A team collaborator who had no contact with participants generated all random assignments, placing them into individual envelopes for use during the data collection. Another member of the study team, who did not participate in data collection, opened each envelope once a participant was successfully enrolled in the study. The procedure ensured that those people directly involved in data collection did not have advance awareness of individual condition assignments. Because of the nature of the study, no one was blinded to the conditions delivered to the participants.

### 2.3 Participants

**Figure 1:**
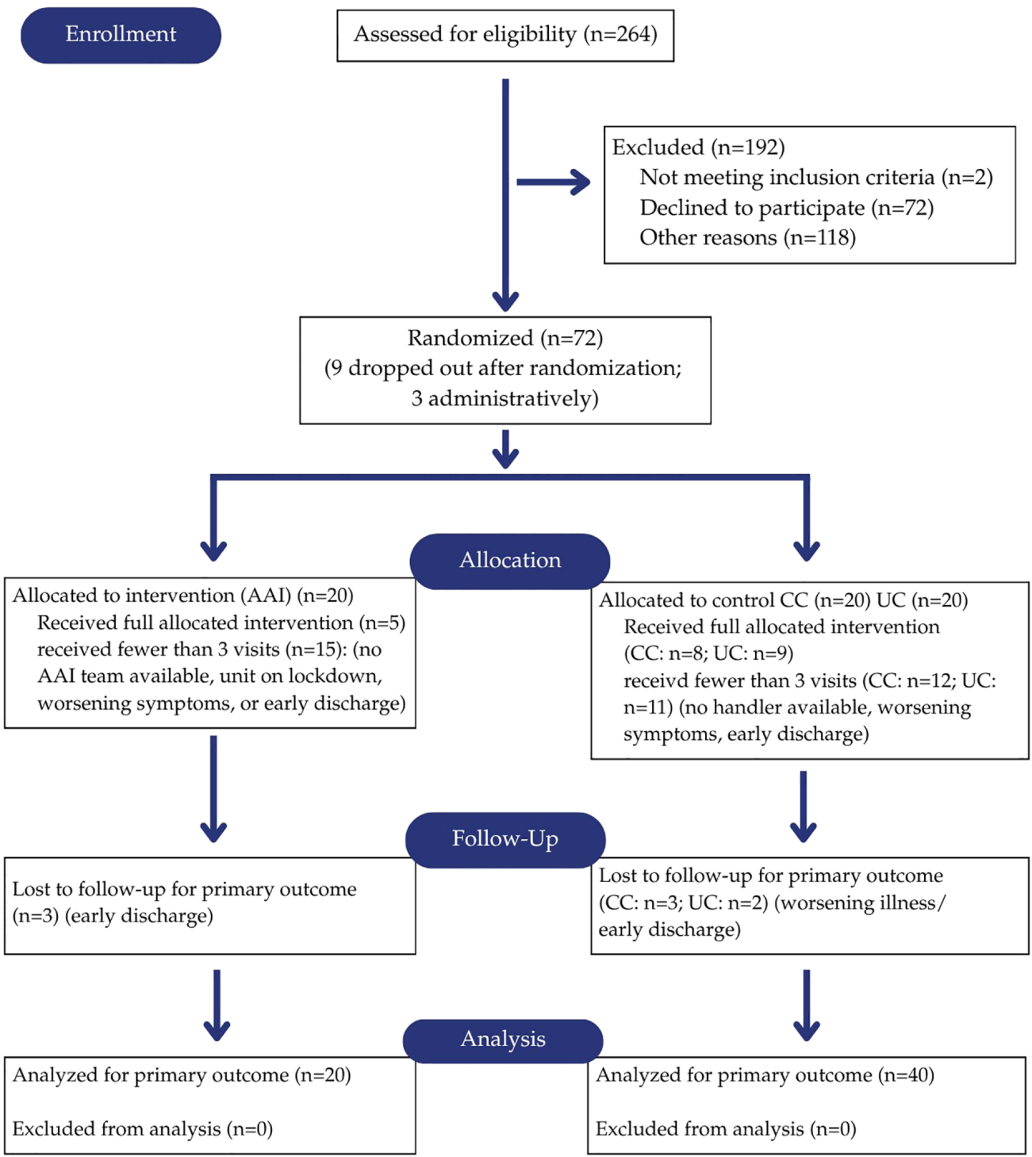
CONSORT diagram (31).

The sample consisted of 60 individuals who were hospitalized and being treated for acute mental illness. Complete demographics of the sample and the individuals randomly assigned to the three intervention groups are included in **Table 1**. A consort diagram for the study is included in **Figure 1**.

**Table 1:** Demographic descriptions of the participants and according to random intervention group assignment: Animal Assisted Intervention (AAI, *n* = 20), Conversational Control (CC, *n* = 20), and Usual Care (UC, *n* = 20).

	All *n*	All %	AAI *n*	AAI %	CC *n*	CC %	UC *n*	UC %	(*df*) χ^2^	*p*
Sex									(4) 5.143	0.273
Female	26	43	8	40	11	55	7	35		
Male	28	47	8	40	8	40	12	60		
Non-binary	6	10	4	20	1	5	1	5		
Race									(6) 7.99	0.238
African American/Black	20	34	4	21	8	40	8	40		
Asian	3	5	2	10	0	0	1	5		
American Indian/Alaskan Native	2	3	0	0	0	0	2	10		
White	34	58	13	68	10	50	11	55		
Ethnicity									(2) 1.067	0.586
Non-Hispanic/Latino	54	96	18	95	19	100	17	95		
Hispanic/Latino	2	4	1	2	0	0	1	2		
Marital Status									(6) 7.466	0.280
Single	31	52	11	55	10	50	10	53		
Married/Partnered	17	29	7	35	5	25	5	26		
Divorced	8	14	2	10	2	10	4	21		
Widowed	3	5	0	0	3	15	0	0		
Education									(8) 2.499	0.952
< High School	4	7	1	5	2	10	1	5		
High School/GED	17	28	6	30	5	25	6	30		
Some College	20	33	8	40	5	25	7	35		
Bachelor’s Degree	11	18	3	15	4	20	4	20		
Graduate Degree	8	13	2	10	4	20	2	10		
Employment Status									(12) 5.815	0.925
Student	3	5.2	1	5	1	5	1	5		
Unemployed (not seeking employment)	10	17	6	32	2	10	2	10		
Unemployed (seeking employment)	17	29	5	29	6	35	6	35		
Self Employed	2	3	0	0	1	5	1	5		
Lives with a Dog									(2) 0.000	1.00
No	39	65	13	65	13	65	13	65		
Yes	21	35	7	35	7	35	7	35		
	All Mean	All SD	AAI Mean	AAI SD	CC Mean	CC SD	UC Mean	UC SD	F (df) =, *p*	
Age (years)	38.7	17.8	36.5	17.4	39.9	18.7	39.6	18.3	(2, 59) = 0.22, 0.80	
BIMS	14.8	0.5	14.8	0.4	14.7	0.7	14.8	0.4	(2, 59) = 0.48, 0.623	
Baseline UCLA-	57.1	11.7	69.4	22.4	66.7	36.5	70.4	22.2	(2, 57) = 1.37, 0.262	
1st Analog Loneliness	68.9	27.4	56.8	10.7	54.2	13.9	60.5	9.5	(2, 59) = 0.08, 0.922	

The sample was approximately equally divided among males (46.7%, n=28) and females (43.3%, n=26) with a smaller number of non-binary participants (10%, n=6). Ages of the participants ranged from 18 to 73 years with a mean of 38.7 (SD = 17.8). Slightly more than half of the participants (52%, n=31) were single/never married, the majority (57.6% n=34) of the sample identified as White and the sample was largely (96%, n =54) non-Hispanic. Twenty-one participants (35.0%) currently lived with a dog. As is demonstrated in **Table 1**, there were no significant differences in demographics between the intervention groups.

### 2.4 Handlers and therapy dogs

There were 16 handlers, of which 15 were female, 15 were white, 1 was white/Hispanic; the average age of those handlers who reported their age (n=12) was 62.50 years. The 16 therapy dogs involved in this study were privately owned and registered therapy dogs who met all requirements of the Center for Human-Animal Interaction Dogs on Call (DoC) program at Virginia Commonwealth University (Center for Human-Animal Interaction, n.d.). DoC is an evidence-based program (23) that has been providing visitation to patients, staff, visitors, and students throughout VCU and the VCU Health System since 2001. See **Table 2** for dog demographics.

**Table 2:** Therapy dog characteristics (N = 16).

Dog	Breed	Gender	Age	Height (inches)	Weight (pounds)
Allie	Miniature Poodle	F	4	15	15
Captain Jack	Shih Tzu	M	9	13	18
Elsa	Golden Retriever/Poodle	F	8	28	50
Hairy Harry	Standard Wire-Haired Dachshund	M	9	10	27
Harry	Golden Retriever/Poodle	M	10	23	55
Homer	Golden Retriever	M	4	29	70
Jag	English Labrador Retriever	M	6	27	100
Maggie	Golden Retriever	F	5	26	60
Matt	Australian Cattle/Border Collie	M	7	22	40
Maxwell	Mixed Breed (Flat-Coated Retriever/Border Collie)	M	7	19	40
Missy	Mixed Breed (Terrier)	F	6	24	60
Murphy	Black Labrador Retriever	M	4	24	100
Phoebe	Maltese/Poodle	F	11	12	5
Roe	Whippet	M	5	22	35
Weird Alf Yank-a-Leash	Mixed Breed (German Shepherd/Aussie/Chow)	M	5	19	50
Wiley	Miniature Schnauzer	M	8	11	7.5

#### 2.4.1 Dog requirements

To participate in the DoC program, all dog-handler teams must hold a current registration with either Pet Partners, or Alliance of Therapy Dogs with an American Kennel Club Canine Good Citizen title. Their adherence to visitation policy and procedures is evaluated in the VCU Health System by program personnel. Dogs must be current on required vaccinations and have yearly veterinary wellness exams that include a negative fecal screening. Handlers are required to keep their dogs clean and odor-free, nails trimmed and smooth, wearing an appropriate collar or harness (no choke, pinch, or e-collars allowed), their DoC vest, and kept on a 4-foot (or shorter) leash.

#### 2.4.2 Handler requirements

All DoC handlers are required to undergo all health and training requirements to be hospital volunteers. This process includes a complete background check and testing for evidence of immunities to a variety of human diseases (e.g., measles). Yearly they are required to receive influenza and COVID-19 vaccinations. Handler trainings, orientations, and observations, include, but are not limited to, HIPAA and Community Engaged Research training (described elsewhere; 32). Handlers are required to wear their DoC polo shirt, VCU Health photo identification card, and their Pet Partners or Alliance of Therapy Dogs identification card during all visits. They must keep their dog’s leash in their hand at all times and carry and use VCU Health approved hand sanitizer that has not expired. Hand sanitizer must be dispensed to participants before and after each session or interaction with the dog.

### 2.5 Measures

We used the Brief Interview for Mental Status (BIMS) as our single participant screening measure in this study (33). The BIMS evaluates attention, orientation, and recall in clinical settings. It has good convergent and divergent validity as well as acceptable reliability (Cronbach’s alpha = 0.77). Intact cognitive functioning was determined by a score of 13 or higher; participants who did not meet this threshold were not eligible for the study. **Table 3** displays the timing of delivery of the measures used in this study.

**Table 3:** Measures used in the study and the timing of the delivery of those measures in the three study phases.

Screening/baseline	Intervention			Post-intervention
Day 1	Day 2	Day 3	Day 4	Day 5
	Pre & Post	Pre & Post	Pre & Post	
BIMS	UCLA-SF	UCLA-SF	UCLA-SF	
Pet Ownership	Analog	Analog	Analog	
UCLA-LS				UCLA-LS

The single screening measure was the BIMS – Brief Inventory for Mental Status. Baseline measures were; Pet ownership history and UCLA-LS – University of California Los Angeles Loneliness Scale (20 item). Intervention measures were administered pre and post intervention; UCLA-SF – University of California Los Angeles Short Form (6-item), and Analog – Analog Loneliness Scale (1-item). The post Intervention measure was the UCLA-LS – University of California Los Angeles Loneliness Scale (20 item).

#### 2.5.1 Demographics

In this study we asked participants to report basic demographic information, including age, gender, race/ethnicity, education level, and employment and marital status.

#### 2.5.2 Pet ownership(s)

We also asked participants about previous and current pet ownership including type and number of companion animal(s). At the 1- and 6-month longitudinal follow ups, we asked, via a 1-item question, about whether there had been any change in the number or type of pets they currently own. Participants could describe any change in pet ownership status in a comment box.

#### 2.5.3 Loneliness

Loneliness was measured on days 1 and 5 and at the 1- and 6-month follow-ups using the 20-item Revised (Version 3) UCLA Loneliness Scale (UCLA-LS) (34). This scale has high reliability (Cronbach’s α = .89 to.94) and has been well-validated across many age ranges. The 6-item UCLA Short Form (UCLA-SF) was used pre/post intervention on days 2, 3, and 4. It has good reliability (Cronbach’s α = .83) and has been validated with student and clinical populations (35). We also used an Analog Loneliness Scale on days 2, 3, and 4, that consisted of one item: “I feel lonely” followed by a 0-100-point visual analog scale illustrated by a horizontal line (36).

### 2.6 Procedure

The study was conducted in an adult general psychiatry inpatient unit at the Virginia Commonwealth University Health’s North Hospital location. There are two adult psychiatric units in the hospital; one designed for patients who may be aggressive and/or experience more extreme psychosis, and the general unit, where this study took place, which includes patients whose symptoms do not prevent them from understanding instructions and who do not pose a significant risk of aggressive behavior.

#### 2.6.1 Recruitment

Clinical staff identified potential patients and study team personnel approached those individuals and provided them with information about the study. Information packets were also made available on the unit. A total of 264 patients were assessed for eligibility. Recruiters were blind to treatment group assignment during enrollment. The participant enrollment rate was 27.3%. The details of recruitment feasibility for this study are reported elsewhere (Townsend et al., under review^1^).

#### 2.6.2 Inclusion/exclusion criteria

*Inclusion criteria* for participants were: English speaking, aged 18+, projected to be admitted to the hospital for the upcoming 5 days, have access to a phone after discharge, and able to provide consent. We assessed their ability to provide consent in three ways: absence of need for guardianship, a BIMS score of 13+, and the clinical judgment of their healthcare team. *Exclusion criteria* were; if patients reported being allergic to or fearful of dogs or were COVID positive or on contact precautions.

#### 2.6.3 Informed consent

We used two stages of digital or written informed consent in this study. In the first stage, participants provided consent for a screening assessment of inclusion/exclusion criteria, including the BIMS. If participants met the inclusion/exclusion criteria they proceeded to the second stage of informed consent to participate in the study. Both stages were completed prior to participants taking part in any aspect of the study.

#### 2.6.4 Participant incentives

Participants were given a $20.00 USD gift card at three different timepoints during the study; 1) upon completion of day 1 measures, 2) upon completion of 1-month follow up measures, and 3) upon completion of 6-month follow up measures. If a participant completed all parts of the study, their total compensation was $60.00 USD.

## 3 Data analysis

Descriptive analyses were conducted prior to hypothesis testing. Demographic characteristics of the three intervention groups were compared with chi squared analyses for categorical variables and one-way ANOVAs for continuous variables.

The first set of inferential analyses addressed changes in loneliness that occurred during the intervention phase. Linear mixed models using the MIXED procedure were used to evaluate changes in UCLA-SF and analog measures of loneliness from day 2 to day 4, changes in UCLA-SF and analog measures of loneliness during (from pre to post) intervention sessions, and differences among the intervention groups in these changes. These were conducted as 2X2 within subjects designs. After checking assumptions, linear mixed models (LMM) with random intercepts (participant ID) were used based on unconditional means intraclass correlations (ICCs) of 0.86 for UCLA loneliness and 0.78 for the analog loneliness scale in the intervention phase of the study. Analyses used all available data with maximum likelihood estimation.

The first set of LMM analyses addressed changes in the intervention phase (days 2 to 4) of the study. Loneliness in this phase was assessed with the UCLA-SF and analog loneliness scores. Predictors in the models examining changes during the intervention phase were: group, representing the three intervention groups; day, representing the first and last days of the intervention phase; and pre-post representing before and after the intervention on each intervention day; and the interactions of intervention group with day and with pre-post, and the three-way interaction of intervention group, day and pre-post. Based on the idea that individuals’ experiences with animals may impact their responses to animals during their hospitalization and concern in the scientific community that women and men might not respond similarly in clinical trials of interventions (37), two sets of exploratory stratified analyses were also conducted: one for individuals who lived with and without dogs at the time of hospitalization and one for females and males. These exploratory analyses were conducted to examine potential unique relationships in these subgroups. They will also provide information to power future studies The hypotheses were tested with the two-way interactions of day and with intervention group and pre-post with the intervention group. Within each set of analyses Bonferroni sequential corrections for multiple comparisons were applied. Estimated means from these analyses were graphed for illustration.

An additional LMM analysis was used to examine differences in UCLA-LS (20 item scale) from baseline to post intervention phase (day 1 to day 5). The loneliness outcome in this analysis was the full UCLA-LS. In this LMM predictors were intervention and time of assessment as well as the interaction of intervention with the time of assessment. The same two sets of stratified analyses as were conducted for the analysis of the intervention period data were also conducted to examine changes from baseline to the day after the intervention. The hypotheses were tested with the interaction of day with intervention group. Sequential Bonferroni comparisons were used for assessment of significance of multiple comparisons as appropriate. All analyses were conducted on an intent-to-treat basis. Return rates on 1 and 6-month follow up measures (e.g., UCLA-LS and change in pet ownership status) were too low to be used in any analyses. SPSS v29 (IBM Corporation, Armonk, NY) was used for all analyses.

## 4 Results

### 4.1 Baseline loneliness

At baseline scores on the UCLA-LS ranged from 20 to 73 (M = 57.1, SD = 11.7). Possible scores on the UCLA-LS range from 20 to 80. The mean UCLA-LS loneliness value was significantly higher and more variable for these psychiatric inpatients than means reported in the instrument validation samples of students, nurses, and older adults (34), which ranged from 31.5 (SD = 6.9) to 40.1 (SD = 9.5).

### 4.2 Pre- to post-intervention (days 2–4) sessions

**Figure 2:**
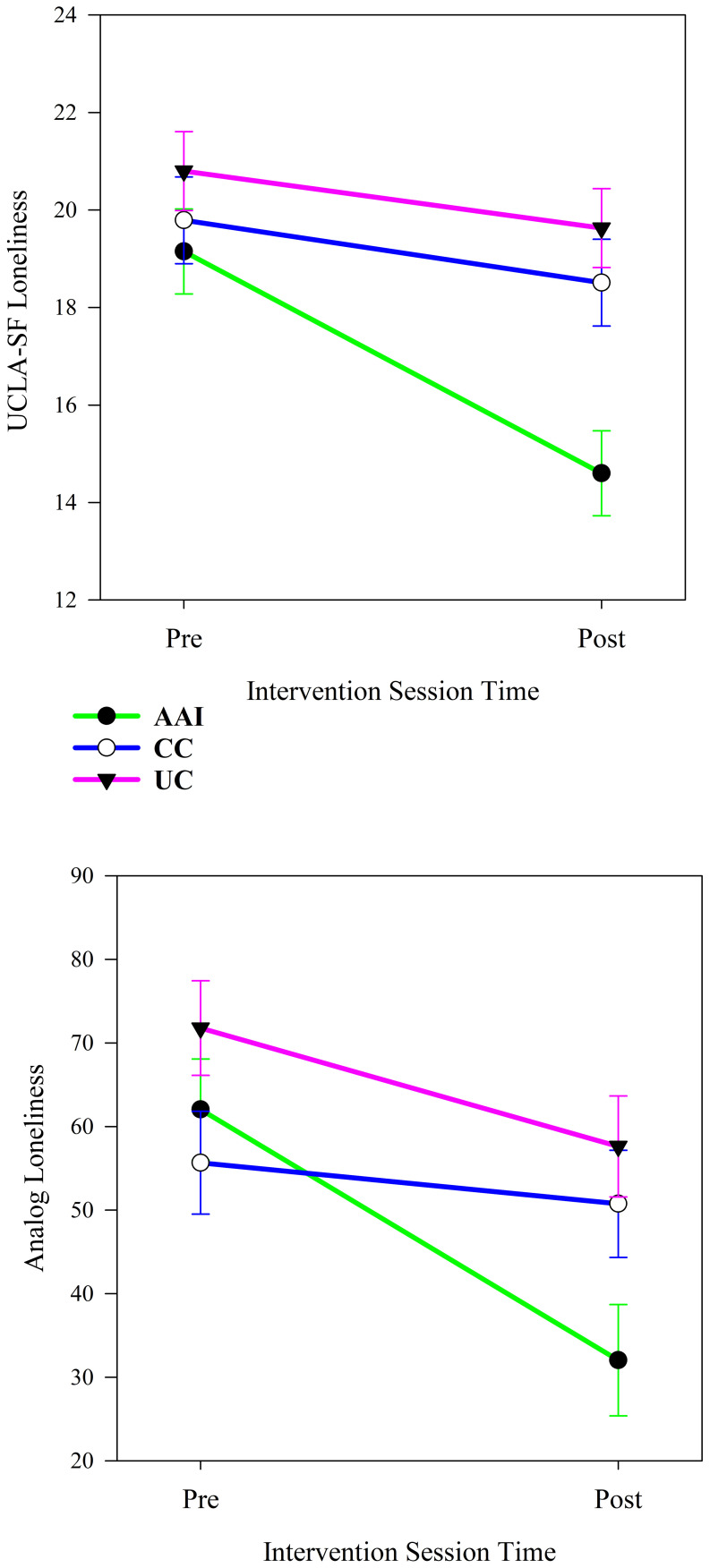
Changes in UCLA-SF and analog measures of loneliness from before to after the intervention sessions according to intervention group (N = 60).

Among all participants, intervention group moderated changes in loneliness from the beginning to the end of the intervention sessions. Intervention groups experienced significantly different changes in both UCLA-SF (*p <* 0.001) and analog scale (*p <* 0.001) measures of loneliness from the beginning to the end of the intervention periods (see **Table 4**). The trajectories of changes in loneliness demonstrated more improvement in both measures of loneliness from before to after the intervention sessions for the AAI than the other groups (see **Figure 2**). The UCLA-SF loneliness scores decreased significantly in all intervention groups (AAI: *p <* 0.001, CC: *p =* 0.002. UC: *p =* 0.002), while the analog loneliness scores decreased significantly in both the AAI and the UC groups (AAI: *p <* 0.001, CC: *p =* 0.212. UC: *p <* 0.001).

**Table 4:** Summary of changes in UCLA-SF and Analog Loneliness scales from the beginning to the end of the intervention phase (days 2–4) of the study and from before to after (pre-post) the intervention sessions according to intervention group: Results of linear mixed models analyses with random intercepts.

	All participants (N = 60)			
	UCLA-SF	ANALOG		
Group (G)	F(2,57.09) = 4.26, *p =* 0.019	F(2,62.57) = 2.47, *p =* 0.09		
Day (D)	F(1,275.75) = 28.15, *p <*0.001	F(1,239.95) = 15.04, *p <*0.001		
Pre-post (PP)	F(1,257.16) = 102.21, p<0.001	F(1,221.15) = 48.87, p<0.001		
D*G	F(2,274.84) = 16.68, *p <* 0.001	F(2,238.37) = 4.42, *p =* 0.011		
PP*G	F(2,257.16) =22.029, p< 0.001	F(2,221.12) =9.30, p< 0.001		
	Females (N = 26)		Males (N = 28)	
	UCLA-SF	ANALOG	UCLA-SF	ANALOG
Group (G)	F(2,522.42) = 3.45, *p =* 0.05	F(2,23.83) = 1.90, *p =* 0.17	F(2,29.92) = 2.25, *p =* 0.12	F(2,31.88) = 0.65, *p =* 0.94
Day (D)	F(1,120.24) = 6.96, *p =* 0.01	F(1,97.337) = 0.63, *p =*0.43	F(1,142.74) = 27.72, *p <*0.001	F(1,128.0) = 11.88 *p <*0.001
Pre-post (PP)	F(1,112.146) = 43.74, p<0.001	F(1,92.766) = 41.36, p<0.001	F(1,126.03) = 61.34, p<0.001	F(1,109.73) = 17.94, p<0.001
D*G	F(2,120.22) = 9.32, *p <* 0.001	F(2,97.35) = 2.57, *p =* 0.08	F(2,137.54) = 11.61, *p <* 0.001	F(2,123.81) = 6.42, *p =* 0.002
PP*G	F(2,112.14) =5.17, p=0.01	F(2,92.75) =13.60, p< 0.001	F(2,126.04) =24.84, p< 0.001	F(2,109.54) =4.68, p= 0.011
	Does not live with dog (N = 39)		Lives with dog- (N = 21)	
	UCLA-SF	ANALOG	UCLA-SF	ANALOG
Group (G)	F(2,57.09) = 4.262, *p =* 0.162	F(2,39.62) = 0.66, *p =* 0.52	F(2,18.379) = 4.32, *p =* 0.029	F(2,19.80) = 3.11, *p =* 0.07
Day (D)	F(1,171.42) = 17.84, *p <*0.001	F(1,152.92) = 25.33, *p <*0.001	F(1,103.44) = 8.30, *p =* 0.005	F(1,80.94) = 0.95, p= 0.33
Pre-post (PP)	F(1,2159.14) = 49.30, p<0.001	F(1,141.32) = 61.66, p<0.001	F(1,90.02) = 60.12, p<0.001	F(1,77.07) = 15.80, p<0.001
D*G	F(2,170.68) = 13.39, *p <* 0.001	F(2,151.88) = 124.41 *p <* 0.001	F(2,103.31) = 4.75, *p =* 0.011	F(2,80.71) = 7.87, *p <* 0.001
PP*G	F(2,2159.14) =11.08, p< 0.001	F(2,141.32) =4.27, *p =* 0.016	F(2,98.02) =13.67, p< 0.001	F(2,77.08) =13.94, p< 0.001

#### 4.2.1 Analyses stratified by sex

**Figure 3:**
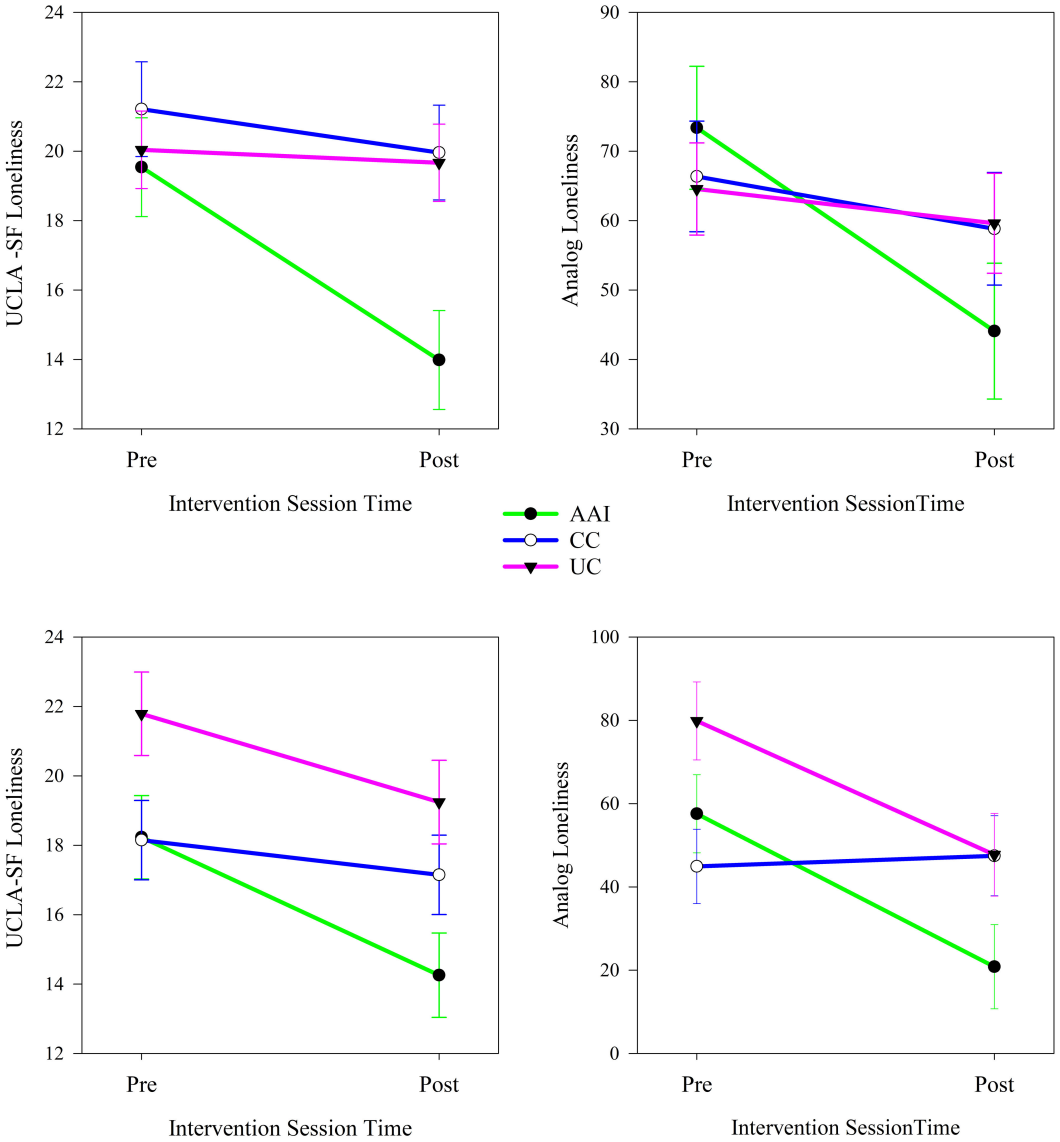
Changes in UCLA-SF and analog measures of loneliness from before to after the intervention sessions according to intervention group for females (top, N = 26) and males (bottom, N = 28).

In analyses stratified by sex, type of intervention moderated changes in loneliness from before to after the intervention sessions. Among both females and males, intervention groups experienced significant changes in both UCLA-SF (females: *p =* 0.01, males: *p <* 0.001) and analog scale (females: *p <* 0.001, males: *p =* 0.011) measures of loneliness from before to after intervention sessions (see **Table 4**). Among females, loneliness assessed with both the UCLA-SF loneliness and analog measures decreased significantly in the AAI and UC intervention groups (all *p*’s < 0.001; **Figure 3**). Among males, UCLA-SF and Analog loneliness decreased significantly from pre to post intervention session in the AAI group (*p’*s < 0.001) and did not change significantly in either other intervention group (see **Figure 3**).

#### 4.2.2 Analyses stratified by dog ownership

**Figure 4:**
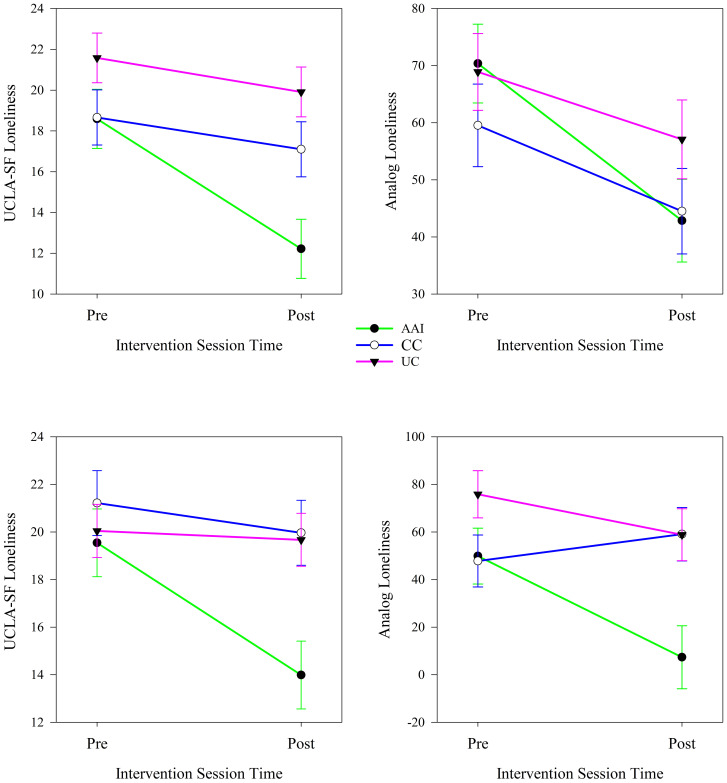
Changes in UCLA-SF and analog measures of loneliness from before to after the intervention sessions according to intervention group for individuals who do not (top, N = 39) and do (bottom, N = 21) live with dogs.

In analyses stratified by dog ownership, intervention group moderated changes in loneliness from before to after the intervention sessions. Among individuals who lived without a dog in their homes, intervention groups experienced significantly different changes in both UCLA-SF (no dog: *p <* 0.001, dog: *p <* 0.001) and Analog scale (no dog: *p =* 0.016, dog: *p <* 0.001) measures of loneliness from pre- to post-intervention sessions (see **Table 4**). Among individuals who did not live with a dog UCLA loneliness decreased significantly from before to after intervention sessions in the AAI (*p <* 0.001) and CC (*p =* 0.015) intervention groups and Analog loneliness decreased significantly in all groups (AAI: *p <* 0.001, CC: *p <* 0.001, UC; *p =* 0.003). The trajectory of change was greater for both loneliness measures in the AAI intervention group than in the other intervention groups (see **Figure 4**). Among individuals who lived with a dog, both loneliness measures decreased differently (*p*’s < 0.001) from before to after intervention sessions according to intervention group (see **Table 4**). Among individuals who lived with a dog UCLA-SF loneliness decreased significantly from before to after intervention sessions in all intervention groups (AAI: *p =* 0.002, CC: *p =* 0.036, UC: *p =* 0.010) and Analog loneliness decreased significantly in the AAI and CC (AAI: *p =* 0.002, CC: *p =* 0.020) groups. The trajectory of change was greater for both loneliness measures in the AAI intervention group than in the others (see **Figure 4**).

### 4.3 Day 2 to day 4: beginning to end of the intervention phase

**Figure 5:**
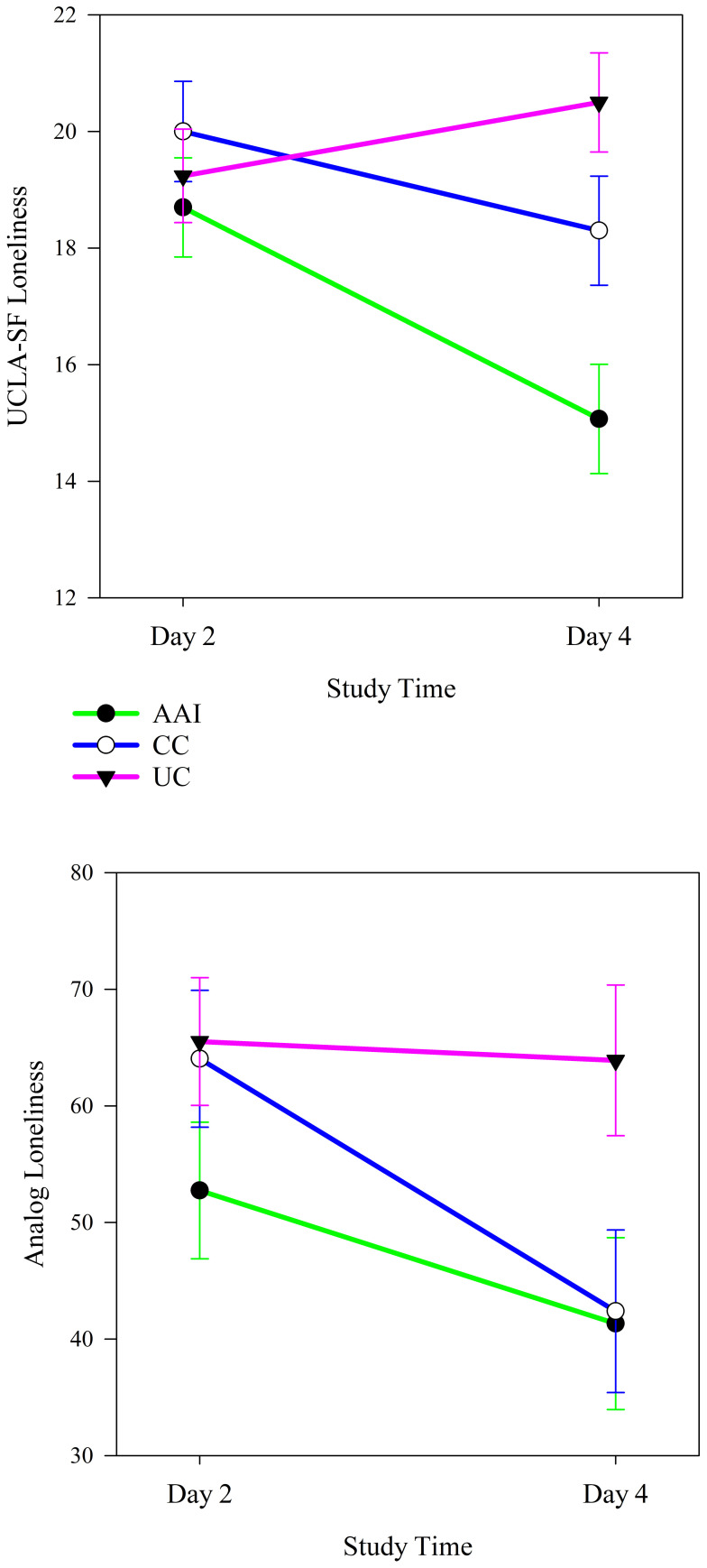
Changes in UCLA-SF and analog measures of loneliness from the first to the last day of the intervention phase according to intervention group (N = 60).

Type of intervention moderated changes in loneliness from the first to the last day of the intervention sessions. Intervention groups experienced significantly different changes in both UCLA-SF and analog scale measures of loneliness from day 2 to day 4 of the study (see **Table 4**) when all participants were considered together. UCLA-SF loneliness decreased significantly from day 2 to day 4 of the intervention in the AAI (*p <* 0.001) and CC (*p <* 0.001) intervention groups; Analog loneliness decreased significantly only in the CC group (*p =* < 0.001). The trajectories of changes in loneliness demonstrated more improvement in UCLA-SF in the AAI group and more improvement in the analog scale of loneliness in the CC than the other groups (see **Figure 5**).

#### 4.3.1 Analyses stratified by sex

**Figure 6:**
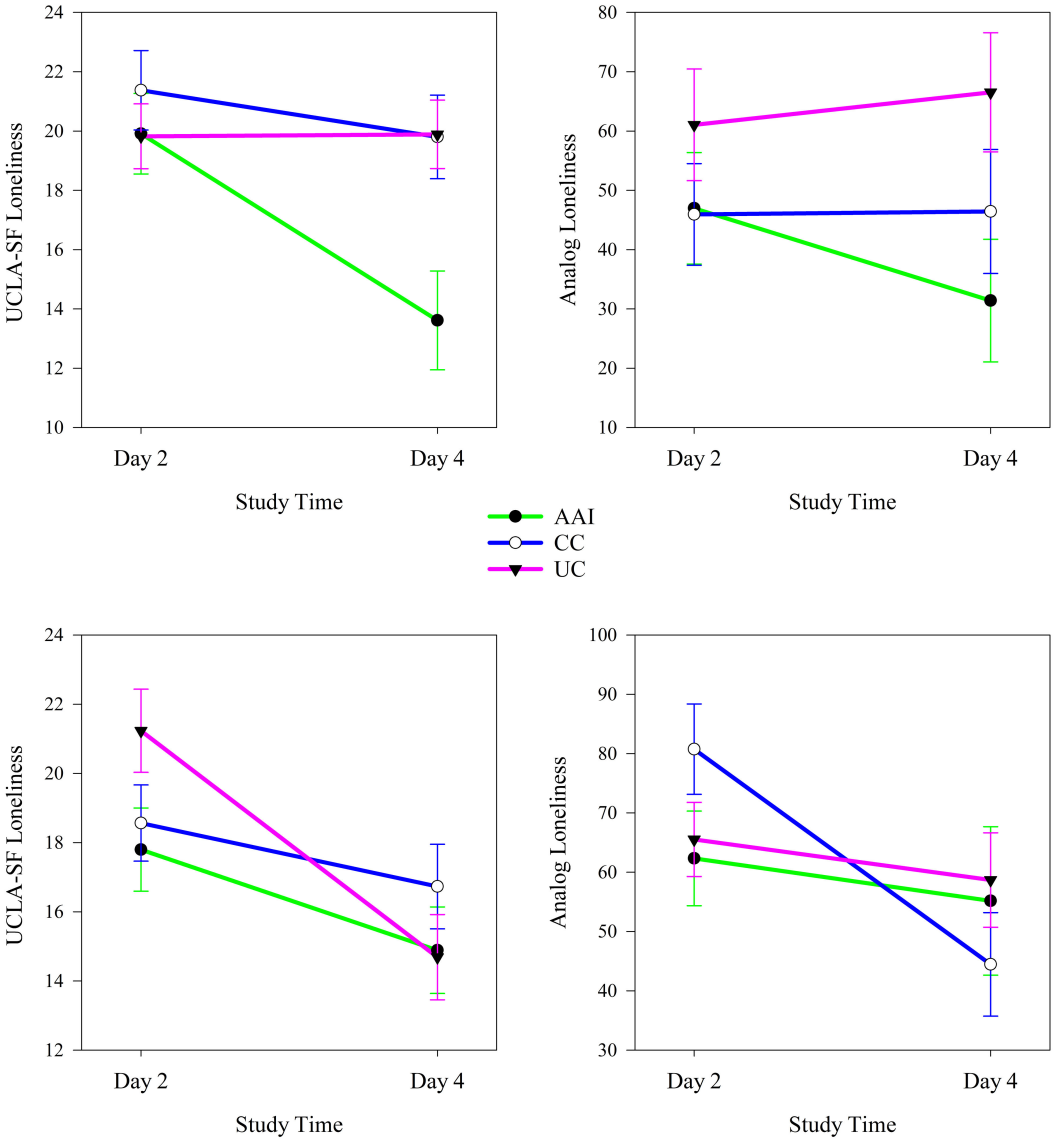
Changes in UCLA-SF and analog measures of loneliness from the first to the last day of the intervention phase according to intervention group for females (top, N = 26) and males (bottom, N = 28).

In analyses stratified by sex, type of intervention moderated changes in loneliness from the first to the last day of the intervention sessions. Among females, intervention groups experienced significantly different changes in UCLA-SF (*p <* 0.001) and among males, intervention groups experienced significantly different changes in both UCLA-SF (*p <* 0.001) and analog scale (*p =* 0.002) measures of loneliness from day 2 to day 4 of the study (see **Table 4**). Among females, the UCLA-SF measure of loneliness decreased significantly from day 2 to day 4 in the AAI (*p <* 0.001) and CC (*p =* 0.017) intervention groups; the Analog measure of loneliness decreased only in the AAI group (*p =* 0.029). Loneliness decreased more in the AAI intervention group than the others (**Figure 5**). Among males, UCLA-SF loneliness decreased significantly from day 2 to day 4 in the AAI (*p <* 0.001) and CC (*p =* 0.015) intervention groups; the Analog measure of loneliness decreased significantly only in the CC group (*p <*.001) and not in the other groups (see **Figure 6**). The small number of non-binary individuals precluded examination of this gender group.

#### 4.3.2 Analyses stratified by dog ownership

**Figure 7:**
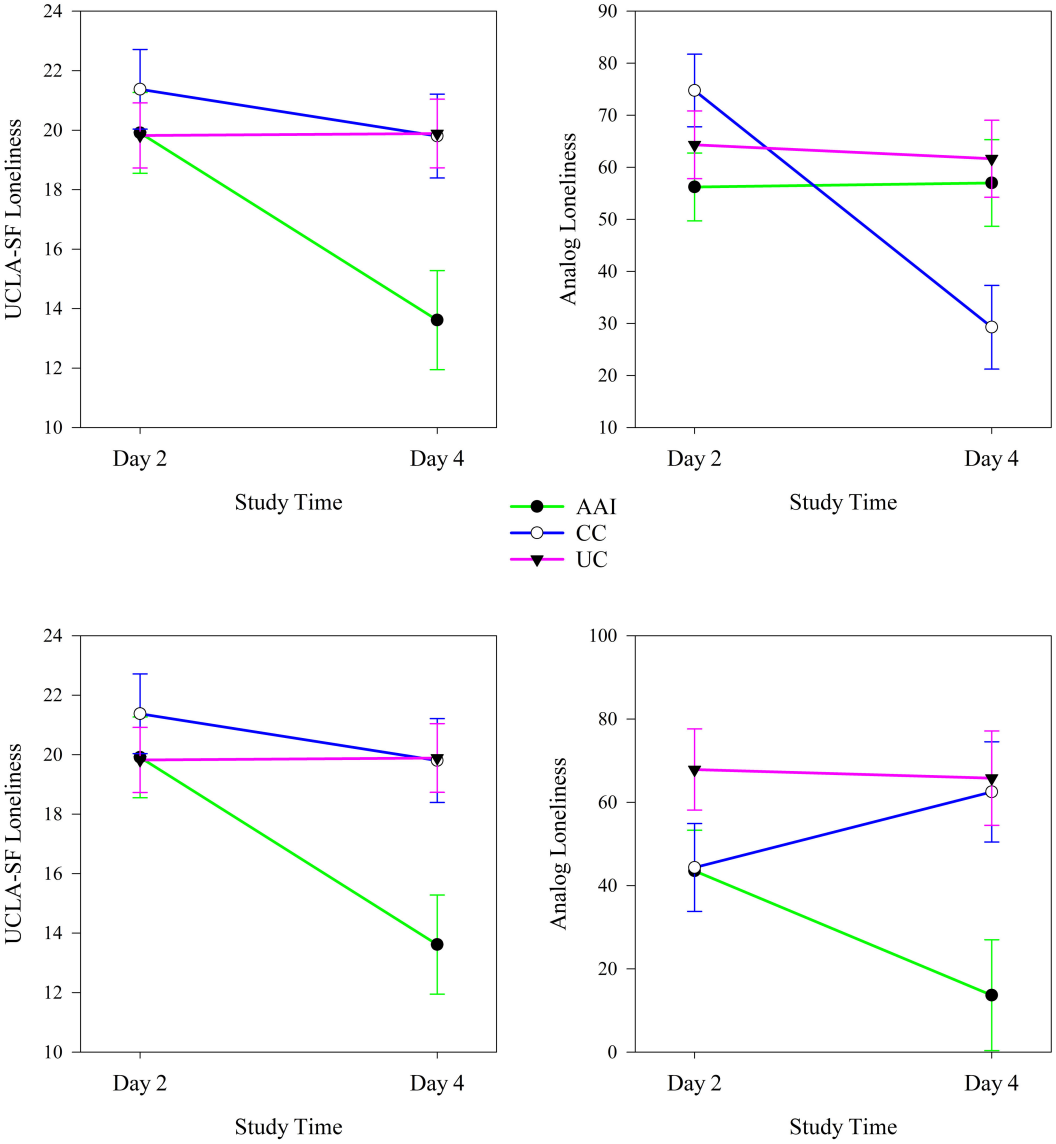
Changes in UCLA-SF and analog measures of loneliness from the first to the last day of the intervention phase according to intervention group for individuals who do not (top, N = 39) and do (bottom, N = 21) live with dogs.

In analyses stratified by living with a dog, intervention type moderated changes in loneliness from the first day to the last day of the intervention (day 2 to day 4 of the study). Among individuals who did not live with a dog, the UCLA-SF measure of loneliness decreased significantly in the AAI (*p <* 0.001) and CC intervention groups while the Analog measure decreased significantly only in the CC (*p =* 0.001) intervention group. Among individuals who did not live with dogs, the UCLA-SF loneliness measure decreased significantly more from the first to the third day of the intervention in the AAI intervention group than in the other intervention groups and the Analog measure of loneliness decreased significantly more in the CC intervention group than in the others (see **Figure 7**). Among individuals who lived with a dog, both the UCLA-SF (*p <* 0.001) and Analog (*p =* 0.002) measures of loneliness decreased significantly in the AAI intervention group over the three days of the intervention. The analog measure of loneliness increased over this same time in the CC (*p =* 0.020) group. Both loneliness measures decreased significantly from the first to the third day of the intervention in the AAI intervention group and not in the other groups (see **Figure 7**).

### 4.4 Day 1 to day 5: baseline to post-intervention

**Figure 8:**
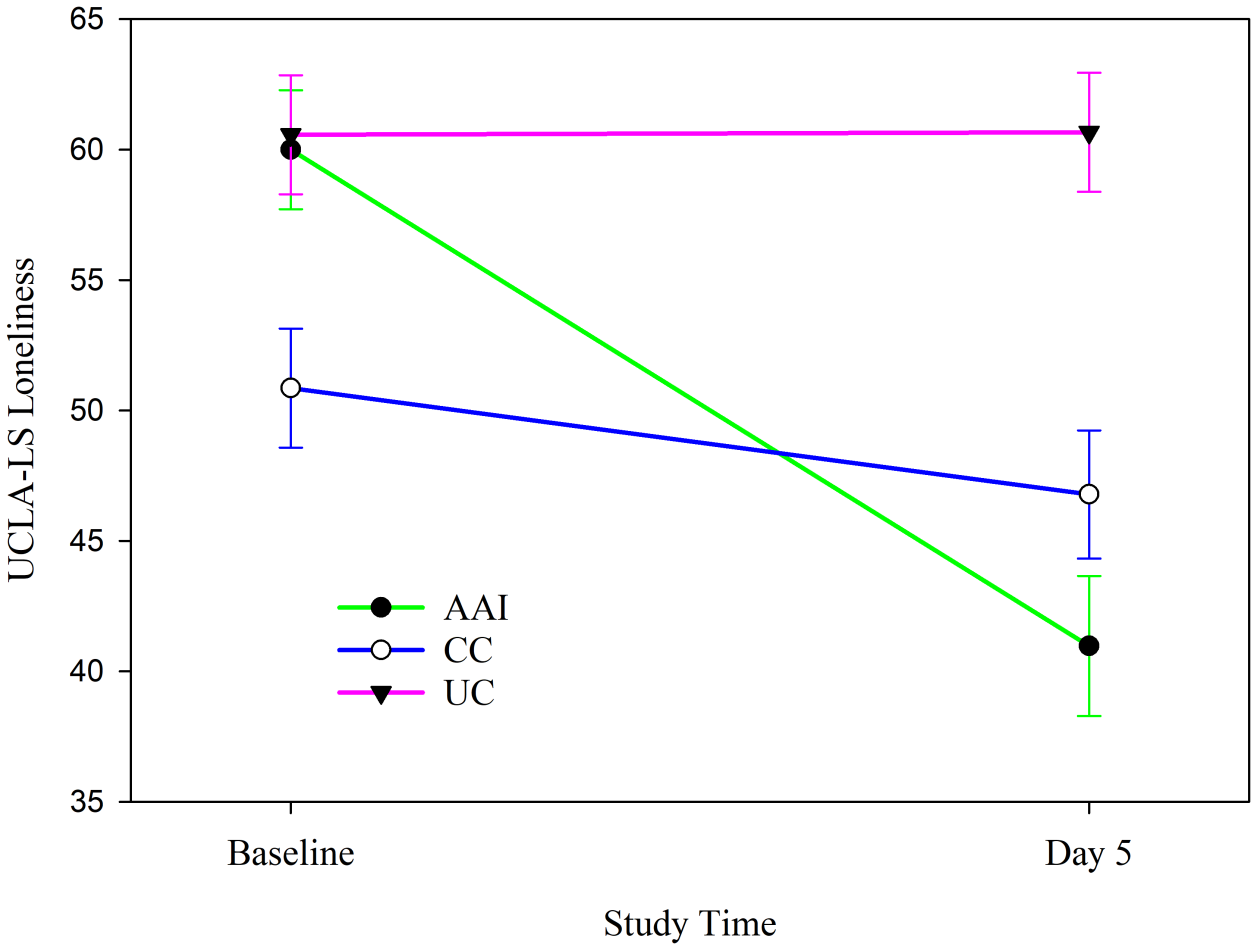
Changes in UCLA-LS loneliness from baseline to the day after the end of the intervention phase according to intervention group among individuals who live with dogs (N = 21).

Among all participants, intervention group did not moderate changes in loneliness (as measured by the UCLA-LS) from baseline to post intervention (see **Table 5**). Stratified analyses revealed that this finding was consistent for males and for females. Similarly, there were no differences according to intervention group in changes in loneliness among individuals who did not have pet dogs at home. The subgroup of individuals who had dogs at home experienced significant differences in changes in loneliness in the three intervention groups (**Table 5**). Dog owners in the AAI group experienced significant decreases in loneliness from Baseline to the post-intervention (*p <* 0.001). Dog owners in the AAI intervention group experienced greater decreases in loneliness over this period than those in other intervention groups (**Figure 8**).

**Table 5:** Summary of changes in UCLA-LS from baseline to the day after the intervention phase (day 1 to day 5) in psychiatric patients according to intervention group: Results of linear mixed models analyses with random intercepts.

	All participants (N = 60)	
Group (G)	F(2,57.82) =4.46, *p =* 0.016	
BL-D5	F(1,54.89) = 6.64, *p =* 0.13	
BL-D5*G	F(2, 54.85) = 1.88, *p =* 0.16	
	Females (N = 26)	Males (N = 28)
Group (G)	F(2,24,82) = 3.89, *p =* 0.034	F(2,26.49) = 0.75, *p =* 0.48
BL-D5	F(1,22.93) = 9.18, *p =* 0.006	F(1,25.67) = 1.09, *p =* 0.31
BL-D5*G	F(2,22.96) = 2.12, *p =* 0.143	F(2,26.62) = 1.04, *p =* 0.37
	Does not live with dog (N = 39)	Lives with dog (N = 21)
Group (G)	F(2.37.27) = 1.25, *p =* 0.30	F(2, 21.48) = 12.44, *p <*.001
BL-D5	F(1, 34.91) = 1.54, *p =* 0.22	F(1, 20.80 = 19.49, *p <*.001
BL-D5*G	F(1,34.91) = 0.14, *p =* 0.87	F(2,20.72) = 10.57, *p <*.001

BL, baseline; D5, day 5 measurement.

## 5 Discussion

The people in our sample, who were hospitalized for the treatment of mental illness, were significantly lonelier than the UCLA-LS instrument validation samples of students, nurses and older adults (34). This is consistent with previous research which indicates that people with mental illness who report being lonely are at increased odds of psychiatric hospitalization, and that loneliness and social isolation represent the two highest-ranked unmet needs in this population (9, 10), Our results showed that although loneliness was improved in all conditions from pre to post daily intervention sessions, the improvement was significantly greater in the AAI condition relative to the other two conditions. This shows that the presence of the dog was responsible for greater immediate improvement in loneliness for psychiatric inpatients. This finding was moderated by sex of patient such that female patients showed improvement in both the AAI and UC conditions whereas male patients only showed improvement in the AAI condition. It is unclear why the females would have reduced loneliness in the UC condition, but it is noteworthy that both sexes experienced a greater reduction in loneliness in the AAI condition, suggesting again that there is something special about the presence of the dog above and beyond that of their handler. When analyzed by dog ownership status, both measures of loneliness improved more in the AAI intervention group than the other two conditions, suggesting that both patients who lived with a dog, and those who did not live with a dog, benefit from the presence of the dog relative to handler only or usual care.

When we examined the results during the intervention phase (days 2–4) from pre to post intervention averaged over each day of the intervention the outcomes were different for the two measures of loneliness. For the UCLA-SF measure, loneliness decreased significantly from pre to post intervention in the AAI and CC conditions relative to the UC condition, suggesting that the dog + handler (AAI) and the handler alone (CC) were both effective in reducing loneliness over those three days. However, the Analog measure of loneliness was only significantly reduced in the handler only (CC) condition and not in the AAI condition. This finding suggests that these two measures of loneliness may be assessing different aspects of loneliness. When the same results were examined by sex, female scores decreased significantly from pre to post for both measures in the AAI condition relative to the other two conditions. Male scores decreased significantly from pre to post for the UCLA-SF in both the AAI and the CC conditions, but the Analog measure only showed a pre to post decrease for the CC condition. Male patients, as measured by the Analog scale, appear to benefit from visits with the handler alone, but it is noteworthy, that in both male and female cases, loneliness, as measured by the UCLA-SF, decreased more in the AAI intervention condition than the other two conditions for both sexes. These results also suggest that the two measures of loneliness may be assessing different aspects of the construct. When these data were analyzed by dog ownership status, loneliness as measured by the UCLA-SF decreased significantly more in the AAI condition than in the other two conditions regardless of dog ownership status. However, the Analog measure again showed different results depending on dog ownership status. For those patients who lived with a dog we saw the same pattern; a significant reduction in loneliness from pre to post, however for those patients who did not live with a dog loneliness was significantly reduced only in the CC condition. Again, these findings suggest that the Analog scale is assessing a different aspect of loneliness than the UCLA-SF scale.

Maes et al. (38) note that many widely used measures of loneliness do not contain language regarding loneliness per se and may measure facets of social connection that contribute differentially to the experience of loneliness. For example, the UCLA contains items assessing both emotional and social loneliness as well as constructs such as extraversion and withdrawal. In comparison, the Analog scale contains one global item (“I feel lonely”) and likely measures what each respondent subjectively defines as “lonely”. The improvement in Analog loneliness for participants only in the conversational control condition might reflect that the focus of the handler was only on the participant and was not shared with the dog. Findings may also reflect scale attributes (the UCLA contains six items and may have tapped loneliness more broadly, whereas the single-item Analog scale may have tapped only a very specific aspect of loneliness that only responded to human-focused interaction).

When we examined the results from baseline (day 1) to follow up (day 5) the reductions in loneliness generally were not maintained, suggesting that the effects are immediate and fleeting. However, it is interesting to note that the dog owners in the AAI group did experience lasting reductions in loneliness relative to the other two conditions suggesting that dog visitation is especially helpful to this group of patients. Transient improvements in loneliness are important because they represent opportunities for people to reduce hypervigilance and dispel cognitive assumptions that inhibit social engagement. Social cognitive theories of loneliness suggest that people may mistrust others based on real or perceived negative experiences and therefore avoid future opportunities to interact and connect (39). Participating in even brief, positive interactions with others offers opportunities to test and dispel deeply rooted beliefs that social interactions are harmful or uncomfortable (40). Dogs are known to be “social lubricants” that increase people’s perception of psychological safety (41), increasing their willingness to connect socially. Positive effects of social interactions involving dogs may also include hormonal shifts that interrupt biological stress responses (41). For those whose mental illnesses consistently isolate them from others, even brief improvements in loneliness and willingness to engage allows them to practice new, positive behaviors that promote future social connections.

Our results replicate and extend the findings of Gee and colleagues (29) with older adults. They found only an immediate, not a lasting, impact of the AAI condition on reducing loneliness for hospitalized older adults. We also found this result, but our results are more nuanced among psychiatric inpatients than those found for older adults. In our case, sex of participant and dog ownership status both played a role in the degree to which the presence of the dog impacts loneliness. The results for both male and female patients were consistent with our expectations that the AAI condition would decrease loneliness, but this loneliness was also decreased for females in the UC condition. It is possible that usual care for female patients is effective at reducing loneliness, but not for male patients. We also found, as expected, that loneliness was reduced in the AAI condition for dog owners and non-owners, but the effect was more apparent for dog owners.

Our results extend those of Gee and colleagues (29) not just because this study examined a group of psychiatric inpatients, but also because our findings were not just immediate in nature, as they were in the Gee et al. study. In that study, only same day pre/post differences were found, but the current study also found reductions in loneliness extending over the 3-day intervention period and for dog-owners the results extended over the 5-day study period. Additionally, the measurement of loneliness resulted in nuanced findings based on which of the two measures were used (UCLA-SF or Analog scale). These findings seem to indicate that these two measures assess different aspects of the construct. The Analog scale is a single item global measure of how lonely a person feels at the moment whereas the UCLA Loneliness Scale has multiple items. This suggestion merits further exploration with a larger sample size to draw definitive conclusions regarding different aspects of loneliness as a measured construct.

### 5.1 Limitations

This study has a number of limitations that mirror those of the Gee et al. (29) study. It used a convenience sample of inpatients, in this case from a psychiatry unit, that may or may not be representative of the larger population of adults hospitalized with mental illness. We elected to recruit a sample of patients based on their need for acute inpatient psychiatric care rather than restrict our sample to those with single psychiatric diagnoses. Although clinical trial research is often conducted with participants who are screened into studies based on uniformity of psychiatric conditions, this does not reflect the patterns of co-morbid psychiatric conditions in the population, placing limits on the generalizability of clinical trial findings (42). Intentionally selecting participants based on uniformity of psychiatric diagnosis would have rendered study findings ungeneralizable given the high rates of co-morbidity among people requiring an inpatient level of care. Although it is possible that the mechanism of effect for AAI is dependent upon or interacts with specific psychiatric diagnoses, we leave the pursuit of that knowledge to future researchers.

The sample size used in this pilot study was too small to make some statistical comparisons that would require dividing the data into even smaller segments (e.g., examining individuals who classified their sex as non-binary). While linear mixed models use maximum likelihood estimation to allow analysis of all available data, the high attrition rates limited the generalizability of null findings about changes from day 1 to day 5 and day 2 to day 4 of the protocol.

We used the same intervention protocol as Gee et al. (29) and it may or may not generalize to other AAI programs, dog-human interactions, or different visitation types or durations. We also did not video-record interactions between patients and dogs, nor did we instruct patients on how they should interact with the dogs. This means that we are not able to comment on whether, or for how long, patients touched or interacted with the dog during the AAI sessions. An informal survey of our handlers indicated that in all cases the patients did touch and interact with the dogs as they typically experience during their normal visitation procedures with other patients in the hospital. For these reasons, we assume that the patients experienced the visitation with the dogs as it is usually delivered in the hospital. The study was not designed to address clustering of dog-handler teams. Participants were not assigned specific teams, so three different teams could visit the same patient during the intervention phase. *Post-hoc*, we examined the distribution of patients across the 16 handler-dog teams who participated in 2–32 session (mean= 7.1, SE = 5.7). We did not include handler as a random effect due to insufficient sample size and convergence issues. This represents a limitation as handler differences could contribute to observed effects. Differences in responses to different teams could be an important component for investigation of the impact of AAI therapy in a larger clinical trial.

## 6 Conclusions

This study offers a number of strengths including the use of a rigorous research design; a randomized controlled trial, replication and extension of previous work, use of a standardized intervention protocol, use of measures with strong psychometric properties, best practice delivery of a dog-visitation process, and high ethical standards for the involvement of both humans and dogs in research. A recent Surgeon General’s Report included six pillars, one of which is to advance social connection and address the loneliness epidemic in the United States and focus on the health sector including assessing and supporting patients (43). The results of the current study replicate and extend those of Gee and colleagues (2024) and support their conclusion that therapy dogs uniquely contribute to the amelioration of loneliness, in this case, in patients with mental illness. Our results also support the potential for AAI as an adjunctive therapy in the treatment of mental illness addressing issues related to loneliness.

## Data Availability

The raw data supporting the conclusions of this article will be made available by the authors, without undue reservation.

## Footnotes

1 Townsend L, Gee NR, Friedmann E, Mueller MK, Thakre TP, Barker SB, (under review). Feasibility of recruiting psychiatrically hospitalized adults for a randomized controlled trial of an animal-assisted intervention.
